# PReS-FINAL-2117: Dream doctors - medical clowns increase the effect of nitrous oxide sedation in intra-articular corticosteroid injection for juvenile idiopathic arthritis

**DOI:** 10.1186/1546-0096-11-S2-P129

**Published:** 2013-12-05

**Authors:** Y Uziel, Y Weintraub, N Rabinowicz, P Hanuka, M Rothschild, S Kotzki

**Affiliations:** 1 Pediatrics, Kfar Saba, Israel; 2 Meir Hospital, Kfar Saba, Israel

## Introduction

Intra-articular corticosteroid injection (IASI), a common procedure in juvenile idiopathic arthritis (JIA), is usually associated with anxiety and pain.

In previous study we concluded that nitrous oxide (NO) provides effective and safe sedation for such procedures. The efficacy in reducing pain was associated with the level of the child's anxiety even before starting the procedure.

Following the introduction of "Dream Doctors" in our hospital, we added medical clown as an important integral part of the team doing IASI.

## Objectives

To prospectively evaluate how a medical clown affects pain perception during IASI in JIA using NO conscious sedation.

## Methods

Patients scheduled for IASI first met and interacted with the medical clown. During the procedure, the rheumatologist and the medical clown worked in parallel to create distraction. NO was administered. The patient, parent, physician, medical clown and nurse completed a visual analog scale (VAS - (0-10) for pain. Change in heart rate (HR) ≥15% was recorded to examine physiologic response to pain and stress.

## Results

46 procedures were performed in 32 children: 23 girls, 9 boys, with a mean age of 10.87 ± 3.58 yrs. The median VAS pain score for the patients, parents, physicians, medical clown and nurses was 2, 2, 1, 1, and 1, respectively. 5 patients had increased HR, and experienced increased pain. In our previous study using only NO, the median pain score was 3.

## Conclusion

Active participation of a medical clown during IASI with NO for JIA further decreasing pain, stress, and induces a pleasant patient experience.

## Disclosure of interest

None declared.

